# Interfacial Characteristics of Ti/Steel Joints Welded by Resistance Spot Welding with Bi-Interlayer of Nb-Ni

**DOI:** 10.3390/ma19122518

**Published:** 2026-06-11

**Authors:** Tong Wu, Xiaowen Li, Yaqiang Wang, Nannan Wang, Ranfeng Qiu, Shengxiong Tang

**Affiliations:** 1 Materials Engineering College, Henan Polytechnic, Zhengzhou 450046, Chinahkdwyq@163.com (Y.W.); 2 School of Materials Science and Engineering, Henan University of Science and Technology, Luoyang 471023, China; 15896555218@163.com; 3 State Key Laboratory of High Temperature Light Alloys and Application Technology, Luoyang 471039, China; 4 Graduate School of Science and Technology, Kumamoto University, Kumamoto 860-8555, Japan; shengxiongtang@163.com

**Keywords:** titanium, steel, resistance spot welding, bi-interlayer

## Abstract

Resistance spot welding was performed to join 2 mm thick TA2 titanium plate and Q235 steel plate using an Nb-Ni bi-interlayer. The microstructure of the interfacial zone was observed and analyzed, and the tensile shear load of the joint was evaluated. The joints obtained display double-nugget-type and penetration-type joints. For the double-nugget-type joint, Ni_6_Nb_7_ and Ni_3_Nb layers have been formed in the region between the residual Nb layer and the steel, while for the penetration-type joint, a mixed nugget composed of Ti-Fe intermetallic compounds was formed at the center zone of the weld. As the welding current increased or welding time extended, the tensile shear load of the joint exhibited a trend of initially increasing and subsequently decreasing. When a bi-interlayer consisting of 0.05 mm-Nb and 0.04 mm-Ni is utilized, the tensile shear load of the joint reached the maximum value of approximately 8.7 kN under the condition of 11 kA welding current, 300 ms welding time, and 3 kN electrode pressure. The results indicate that the Nb layer can effectively impede the cross-interface diffusion of Ti atoms and Ni-Nb intermetallic compound layers are formed in the interface region in the case that the interlayer thickness and the welding parameters are well-matched when resistance spot welding of Ti/steel is performed with a bi-interlayer of Nb-Ni.

## 1. Introduction

In the structural design of the product, employing corresponding materials for components with different performance requirements can not only fully leverage the respective advantages of the materials but also reduce the manufacturing cost [1]. Titanium and its alloys exhibit high strength, low density, and excellent corrosion resistance, whereas steel possesses good processing properties and a low cost [2,3]. The design of the titanium–steel bimetallic structure can broaden the application scope of the relatively costly titanium [4,5]. For instance, the Q235 steel chassis frame and the titanium alloy guard plate within the battery pack frame of an automobile, as well as the Q235 profiles and titanium sheets in the interior framework of an aircraft cabin, all require joining. However, this structure requires an effective connection between titanium and steel to offer support. Due to the significant differences in physical and metallurgical properties between titanium and steel, when titanium and steel are welded, not only can brittle Ti-Fe intermetallic compounds (IMCs) be formed, but also a substantial amount of stress is generated at the welding interface zone [6,7]. To mitigate the impact of interface IMCs, certain solid-state welding techniques, including friction welding [8], friction stir welding [9], explosive welding [10], ultrasonic welding [11], and diffusion welding [12,13], are employed for the welding of titanium and steel. The interfacial structure and properties of the joints welded using the solid-state welding techniques have also undergone extensive research. Nevertheless, the interfacial IMCs continue to be a crucial factor constraining the performance of the joints, and the welding process for titanium/steel necessitates further optimization [14].

For the welding of dissimilar materials, applying an interlayer to inhibit the growth of interfacial IMCs or to form a less brittle IMCs instead of the more brittle Ti-Fe-type IMCs by regulating the interfacial metallurgical reaction during the welding process is considered an acceptable option [15]. Zhang et al. [16] employed a Ta-V-Fe composite interlayer to direct the laser beam towards the titanium side and conducted laser welding on TC4 titanium alloy and 301L stainless steel. Kundu et al. [17] employed a Ni–17Cr–9Fe alloy as a transition layer to perform diffusion welding on TC4 and duplex stainless steel. The tensile strength, elongation, and shear strength of the resultant joints were increased by 16.1%, 30.6%, and 12.1% respectively, in comparison with the joints without the application of a transition layer [17]. Zhang et al. [18] conducted an investigation into the influence of the inclusion of Cr and V powder layers on the resistance brazing of TC4 and 304 steel. Previous research findings have indicated that the incorporation of the interlayer can effectively suppress the growth of Ti-Fe IMCs at the interface, thus enhancing the joint performance. In the case of thin-plate lap welding, which is commonly employed in resistance spot welding (RSW), owing to the local melting of the base material during the welding process, a substantial amount of Ti-Fe IMCs can be formed in the direct resistance spot welded (RSWed) joint between titanium and steel, leading to suboptimal joint performance [19].

The interlayer for RSW of dissimilar materials is typically selected based on the properties of the two materials to be welded. In previous studies, a preliminary investigation was carried out by selecting Nb foil as the interlayer in accordance with the characteristics of titanium [20]. The research findings indicate that despite the fact that utilizing Nb foil as the interlayer for RSW can effectively inhibit the diffusion of Ti into the steel, the joint failure observed in the tensile shear test predominantly took place at the interface between the Nb layer and the steel [20]. On the other hand, when Ni foil was utilized as the interlayer for RSW between titanium and steel taking into account the characteristics of steel, it was discovered that the bonding between the interlayer Ni and steel is relatively sound [21]. However, at the interface between the interlayer Ni and titanium, owing to the formation of Ni-Ti IMCs, this interface became a weak point of the joint [21]. In this study, therefore, taking into account the base material properties of both titanium and steel, a Nb-Ni bimetal was proposed as the interlayer for RSW of Ti/steel, and the phenomenon at the Nb/Ni interface within the resulting joint was investigated for the first time. The purpose of the study is to study the features of the formation and characteristics of joints between titanium and steel during RSW using a bi-interlayer of Nb-Ni, as well as to determine the optimal parameters of the welding process to achieve maximum joint strength.

## 2. Experimental Materials and Procedures

In this study, the materials intended for welding are 100 mm × 30 mm × 2 mm TA2 pure titanium sheet and Q235 low-carbon steel sheet. Their compositions are presented in Table 1.

Nb foil and Ni foil were selected as the interlayers. The thickness of the applied Ni foil was kept constant at 0.04 mm, while the Nb foils used had three different thicknesses: 0.02 mm, 0.05 mm, and 0.10 mm. These interlayers were processed to meet a specification of 30 mm × 30 mm. The surfaces of the base materials and interlayers were cleaned with anhydrous ethanol and then dried. Prior to RSW, the TA2 titanium plate and the Q235 low-carbon steel plate were overlapped with an overlap length of 30 mm. Meanwhile, the Nb foil and the Ni foil were placed between them, with the Nb foil adjacent to the titanium side and the Ni foil adjacent to the steel side.

**Figure 1:**
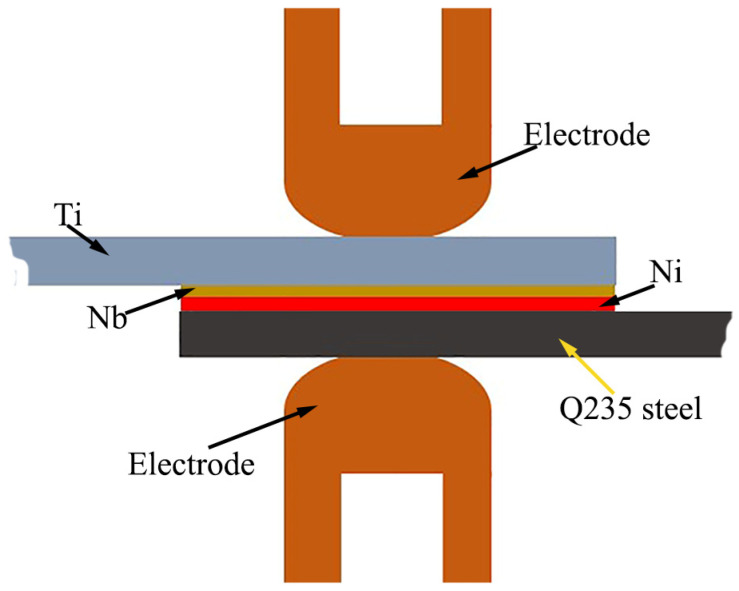
Schematic diagram of Ti/steel RSW with Nb-Ni bi-interlayer.

The RSW was carried out using the DM-200 medium-frequency inverter RSW machine (DM200, Medar, Shanghai, China). The titanium plate was placed on the upper electrode side, and the steel plate was placed on the lower electrode side as shown in Figure 1. The electrode employed had a tip diameter of 6 mm and was fabricated from CrZrCu alloy. During RSW, the electrodes were precisely aligned with the center of the overlapping area of the sample welded.

The detailed welding conditions are presented in Table 2 and Table 3. In the Nb-Ni bi-interlayer, the Nb foil with a high melting point primarily functions as a barrier. The thicker the applied Nb foil, the higher the welding current needed for the rupture of the Nb layer during the welding process [20]. Therefore, when conducting welding operations with varying welding currents, the range of welding currents selected varies according to the different thicknesses of the interlayer Nb foil as presented in Table 2. When the welding time is changed during the welding process, the value of the selected welding current is determined based on the performance of the joint obtained by changing the welding current as presented in Table 3. Additionally, the squeeze time (refers to the duration during which the electrode pressure is applied before the activation of the welding current) and the holding time (denotes the duration during which the electrode pressure is maintained after the welding current is turned off) were both set to 200 ms.

For each set of parameters, seven joints were welded. Among these joints, five were utilized for the tensile shear load test, and two were employed for observing the microstructure of the joint. Following the RSW process, the joints were cut along the diameter of the weld and perpendicular to the welding interface. Subsequently, the cross-sectional surface of the obtained joint was to be ground and polished. A scanning electron microscope (SEM, GeminiSEM 360, Zeiss, Cambourne, UK) equipped with an energy dispersive spectrometer (EDS, Ultim Extreme, Oxford, UK) and electron backscatter diffraction (EBSD, C-Nano, Oxford Instruments, Oxford, UK) device was employed to observe the microstructure of the joint and perform component analysis on specific areas. When performing SEM observations on the cross-section of the joint, the backscatter mode was utilized. EBSD specimens were prepared by polishing with argon ions. The step size, working distance, and accelerating voltage, and specimen tilt angle for EBSD measurement were 0.15 μm, 15 mm, 20 kV, and 70° respectively. Pole figures (PHs) were generated by equal-area projection after data smoothing. All EBSD data were analyzed by using AZtecLive 6.2 SP1. The overall indexing rate was maintained above 90%. Utilizing a tensile testing machine (AG-1205 kN, Shimadzu Corporation, Shimadazu, Japan), a tensile shear test was carried out on another portion of the joints at room temperature at a rate of 1.0 mm/min. The fracture patterns of the joints were observed and analyzed.

## 3. Results and Discussion

**Figure 2:**
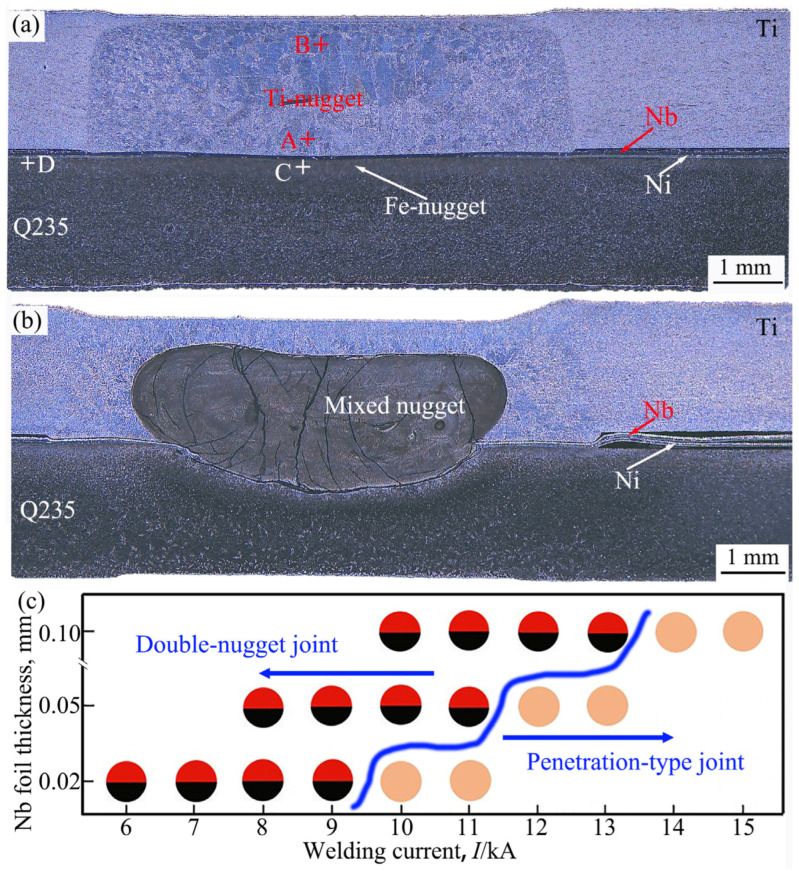
Cross-sections of Ti/steel joint and effect of welding current on nugget pattern for different Nb interlayer thickness patterns: (**a**) double-nugget-type; (**b**) penetration-type; (**c**) the relationship between the nugget pattern and the welding conditions.

Figure 2a and Figure 2b depict the cross-sections of the joints between TA2 pure titanium and Q235 steel (hereinafter referred to as Ti/steel joints), which were fabricated by RSW using a bi-interlayer consisting of a 0.05 mm-thick Nb foil and a 0.04 mm-thick Ni foil under currents of 11 kA and 12 kA, respectively. As shown in Figure 2a, a nugget was observed on both the titanium side and the steel side of the joint, which are respectively referred to as Ti-nugget and Fe-nugget. In the Ti/steel joint, the Ti-nugget and the Fe-nugget are separated by the interlayer, and no penetration occurs between them. Joints with this nugget pattern are referred to as double-nugget-type joints in this study. As depicted in Figure 2b, a trans-interface nugget was observed in the joint welded under a higher welding current condition, and this nugget is termed a mixed nugget. At the mixed nugget, the titanium and steel on both sides of the joint were mixed during the welding process. In this study, joints featuring a mixed nugget pattern are referred to as penetration-type joints. In addition, a substantial number of cracks were observed within the mixed nugget, and these cracks were approximately perpendicular to the welding interface. Under the current magnification, approximately 30 cracks were detected within the mixed nugget. The longest crack, measuring approximately 2 mm in length, penetrated vertically through the mixed nugget. During the cooling process of welding, the molten nugget region was subjected to tensile stress as it solidified and contracted, being constrained by the surrounding solid base material. Under tensile stress, crystallization cracks were formed within the nugget during the latter half of solidification. Since the mixed nugget is predominantly composed of hard and brittle Ti-Fe MCs (as elaborated below), cracks predominantly were formed within the mixed nugget. The application of electrode force persisted throughout the solidification stage of the nugget, which mitigated the tensile stress endured by the nugget in the direction perpendicular to the welding interface. Consequently, the nugget predominantly bore tensile stress parallel to the welding interface. In addition, the mixed nugget mainly consists of coarse columnar crystals that are oriented approximately perpendicular to the welding interface (details are provided below). Therefore, the cracks that formed mostly propagated in a direction approximately perpendicular to the welding interface.

Observation results indicate that the mode transition of the Ti/steel joint is related to the welding conditions. Figure 2c demonstrates the impact of welding current on the joint mode. When the thicknesses of the Nb foils employed were 0.02 mm, 0.05 mm, and 0.10 mm, the joints obtained under welding currents greater than 10 kA, 12 kA, and 14 kA, respectively, were penetration-type joints. That is to say, the thicker the Nb foil employed, the higher the critical welding current at which the joint transitions from the double-nugget-type to the penetration-type.

For both types of joints, the size of the nugget on the titanium side is greater than that on the steel side. This is due to the fact that the resistivity of TA2 titanium is greater than that of Q235 steel. During the electrification process, more heat is generated on the TA2 titanium side than that on the Q235 steel side. Furthermore, the thermal conductivity of TA2 titanium (21.9 W·m^−1^·K^−1^) is lower than that of Q235 steel (approximately 82.0 W·m^−1^·K^−1^). In essence, during RSW, the TA2 titanium side generated more heat and dissipated less heat compared to the Q235 side.

**Figure 3:**
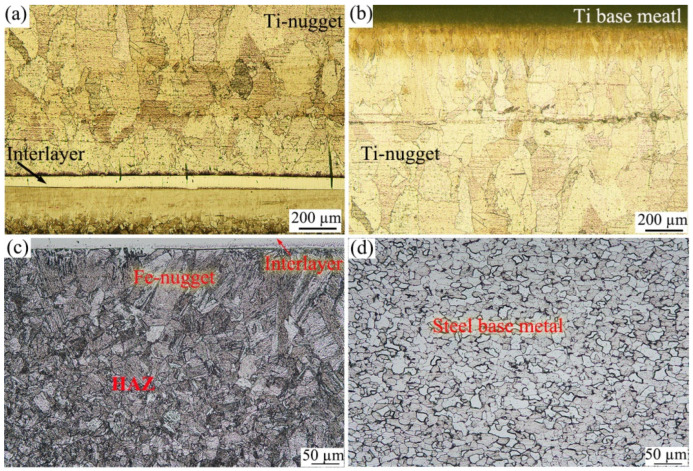
Metallographic images of the nugget zone of the double-nugget-type joint: (**a**) lower boundaries of the Ti-nugget; (**b**) upper boundaries of the Ti-nugget; (**c**) Fe-nugget; (**d**) steel base material.

Figure 3 shows the metallographic images of the nugget zone of the double-nugget-type joint. Figure 3a and Figure 3b display the images at the lower and upper boundaries of the Ti-nugget, respectively, taken from points A and B in Figure 2a. The interlayer (Nb layer, details provided below) was observed beyond the lower boundary of the Ti-nugget. This suggests that the Nb layer applied during the welding process remained in a solid state and functioned as a barrier to prevent the mixing of the base materials on both sides. In comparison with the coarse columnar crystals in the central region of the Ti-nugget, there was a band-shaped region with relatively smaller grains, approximately 200 µm in thickness, adjacent to the Nb layer. Likewise, a band-shaped region with smaller grains also existed in the vicinity of the TA2 base material at the upper boundary of the Ti-nugget. Figure 3c and Figure 3d respectively show the metallographic photos of the Fe-nugget and the steel base material zone, which were respectively taken from points C and D in Figure 2a. The grains in the Fe-nugget, which is close to the interlayer, are significantly coarser than those in the base material area. Moreover, the Fe-nugget is primarily composed of ferrite. Beyond the Fe-nugget zone, the heat affected zone (HAZ) was detected. This zone was primarily composed of fine ferrite, pearlite, and coarse ferrite. Owing to the inadequate welding heating within this zone, there was incomplete austenitization of some ferrite, leading to the formation of an uneven mixed structure upon cooling.

**Figure 4:**
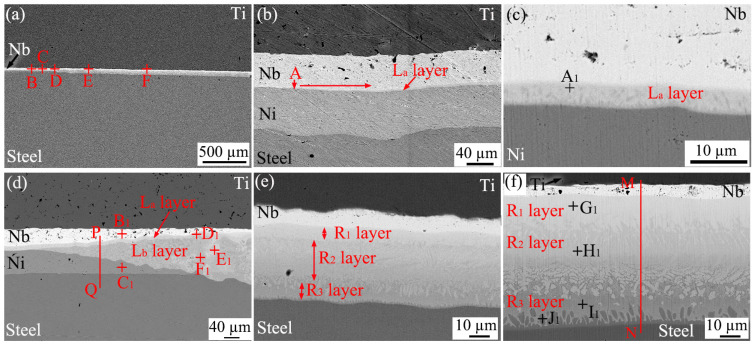
Interfacial SEM images of joint with double-nugget-type joint: (**a**) low-magnification SEM image; (**b**–**f**) enlarged image of B~F locations, respectively.

Figure 4 presents the SEM images of the interface region of the double-nugget-type joint. As depicted in Figure 4a, a continuous interlayer was detected in the interface region. Figure 4b, Figure 4c, Figure 4d, Figure 4e and Figure 4f are the magnified images of points B, C, D, E, and F in Figure 4a, respectively. EDS analysis was carried out on the characteristic regions, and the results are presented in Table 4. As depicted in Figure 4b, a band-shaped reaction layer (L_a_) was observed to be formed at the interface between Ni and Nb starting from the outer edge (point A) of the weld. As the position approaches the weld center, the formed L_a_ layer gradually thickens. At the interface at point C, as shown in Figure 4c, the thickness of the formed L_a_ layer is approximately 5 μm. The results of the component analysis indicate that Ni and Nb were detected in the L_a_ layer (location A_1_), whereas Ti and Fe were not detected. This layer primarily consists of Ni_3_Nb and Ni_6_Nb_7_.

**Figure 5:**
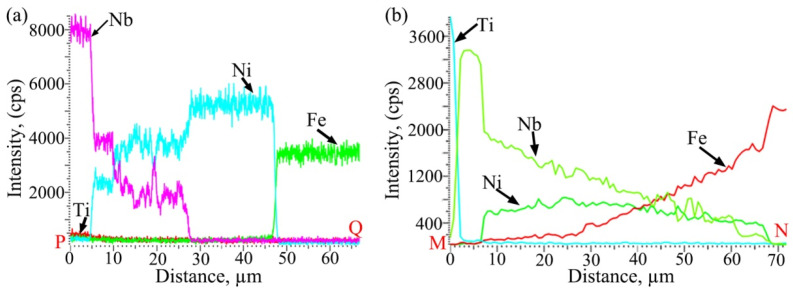
EDS results: (**a**) along PQ in Figure 4d; (**b**) along MN in Figure 4f.

As the center of the welding zone is approached, a transition zone with a gradually varying thickness of the interlayer (Nb layer and Ni layer) was observed at point D, as depicted in Figure 4d. In this zone, the Nb layer and Ni layer gradually thinned, and the Ni layer even disappeared completely, while the L_a_ layer not only persisted but also formed a new reaction layer, the L_b_ layer. The thickness of the L_b_ layer increased as the Nb layer and Ni layer on both sides thinned. EDS analysis was carried out along PQ in Figure 3d, and the results are presented in Figure 5a. Compared with the L_a_ layer, the Ni content detected in the newly formed L_b_ layer is higher, whereas the Nb content is lower. A small quantity of Ti and Ni was detected in the residual Nb layer in this area, and a small quantity of Fe was detected in the residual Ni layer. This is the outcome of atomic inter-diffusion in the interface region; however, no Nb was detected in the residual Ni layer. The detection results at the L_a_ layer (D_1_ position) in this section indicate that its composition remains a mixture of Ni_3_Nb and Ni_6_Nb_7_ phases. As depicted in Figure 4d, within the L_b_ layer, there exist two regions with distinct contrasts (a light-gray region and a dark-gray region), which are interlaced and randomly distributed. The analysis results of the light-gray region (at location E_1_) and the dark-gray region (at location F_1_) indicate that the former primarily consists of Ni_3_Nb and Ni_6_Nb_7_, while the latter mainly comprises (Ni) and Ni_3_Nb. The L_b_ layer was formed via the melting of the Ni layer during the welding process, the dissolution and mixing of the adjacent Nb into it, and the subsequent solidification.

During the stage where the entire Ni layer has melted, the adjacent steel also dissolved into it. As depicted in Figure 4d, the L_b_ layer near the steel displays a greater thickness, while the L_a_ layer adjacent to the Nb layer shows a lesser thickness. As previously mentioned, the reason can be attributed to the higher peak temperature and longer high-temperature dwell time on the Ti side. During the cooling process, the mixed liquid metal solidified, resulting in the formation of IMCs. Given that this area is situated at the edge of the weld zone and has a shorter high-temperature dwell time, the liquid metal did not mix thoroughly. Nb was distributed unevenly in the liquid metal, and the types of IMCs formed after solidification also differ. In the area with a relatively higher Nb content (location E_1_), Ni_3_Nb and Ni_6_Nb_7_ were formed; in the area with a relatively lower Nb content (location F_1_), (Ni) and Ni_3_Nb phases were formed.

As one approaches the weld center, the reactants in the interface zone gradually form a layered structure, as shown in Figure 4e. This structure comprises the R_1_ layer adjacent to the residual Nb layer, the R_3_ layer adjacent to the steel side, and the R_2_ layer situated between them. The closer to the weld center, the thicker these reaction layers become. As depicted in Figure 4f, in the vicinity of the welding center, the thicknesses of layers R_1_, R_2_, and R_3_ attain approximately 16 μm, 26 μm, and 15 μm respectively. Based on the EDS results, it can be deduced that the primary constituent phases of the R_1_ and R_2_ layers are Ni_6_Nb_7_ and Ni_3_Nb, respectively. The R_3_ layer consists of a mixture of (Fe) and Ni-Nb IMCs. Significantly, a minor quantity of Ti was detected in all the R_1_, R_2_, and R_3_ layers, suggesting that when the residual Nb layer is extremely thin, a small number of Ti atoms can diffuse through the Nb layer to the opposite side. As depicted in Figure 4f, within the R_3_ layer adjacent to the steel side, the subsequent blocky products were detected. The composition analysis (at location J_1_) revealed that these products were solid solutions of Ni in Fe. A line analysis was carried out along MN in Figure 4f, and the corresponding results are presented in Figure 5b. These results demonstrated that the content of Fe gradually rose in the R_1_, R_2_, and R_3_ layers. During the welding heating process, Ni and the adjacent steel underwent melting and mixing. Following the cooling and solidification of the mixed liquid composed of Ni, Nb, and Fe in the interface zone, a layered structure was formed. Owing to the slight inter-mixing of Fe and the dissolution of Nb, the contrast of the interface layer was altered.

**Figure 6:**
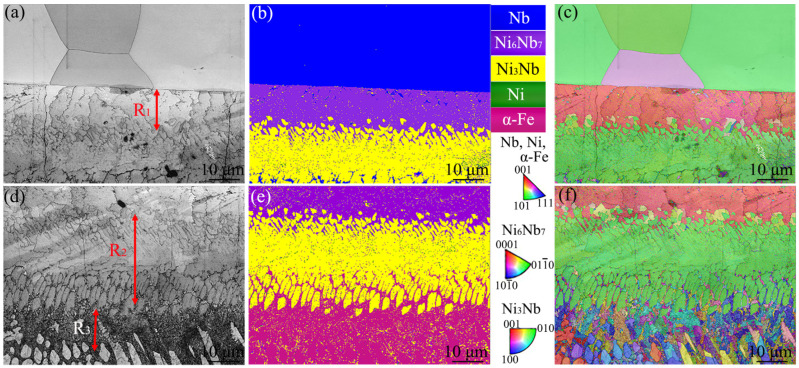
EBSD results in interfacial reaction zone near weld center: (**a**–**c**) SEM image, phase distribution and grain orientation distribution adjacent to the Nb layer; (**d**–**f**) SEM image, phase distribution and grain orientation distribution adjacent to the steel.

Figure 6 showcases the EBSD detection results of the interface zone in the vicinity of the weld center. Specifically, Figure 6a, Figure 6b and Figure 6c respectively depict the SEM image, phase distribution, and grain orientation distribution of the zone adjacent to the Nb layer; while Figure 6d, Figure 6e and Figure 6f respectively illustrate the SEM image, phase distribution, and grain orientation distribution of the zone adjacent to the steel side. It was further confirmed that the R_1_ layer adjacent to the residual Nb layer primarily consists of Ni_6_Nb_7_, whereas the R_2_ layer adjacent to the steel side primarily consists of Ni_3_Nb. Furthermore, as can be observed from Figure 6e, the R_3_ layer consists of α-Fe and a minor quantity of Ni_3_Nb. The Ni_3_Nb and Ni_6_Nb_7_ IMCs exhibit relatively high hardness, with hardness values of 330 HV and 450 HV respectively [22]. The thickness of the Ni_6_Nb_7_ layer and Ni_3_Nb layer are approximately 14 μm and 25 μm, respectively. The grain size of the Ni_6_Nb_7_ and Ni_3_Nb phases exhibits non-uniformity in the zone. The arithmetic mean of the equivalent circular diameter of the Ni_6_Nb_7_ grains is 1.03 μm. The maximum equivalent circular diameter of these grains is approximately 14.61 μm, and the standard deviation is 1.04 μm. On the other hand, the arithmetic mean of the equivalent circular diameter of the Ni_3_Nb grains is 0.96 μm, with a maximum value of 15 μm and a standard deviation of 0.85 μm.

**Figure 7:**
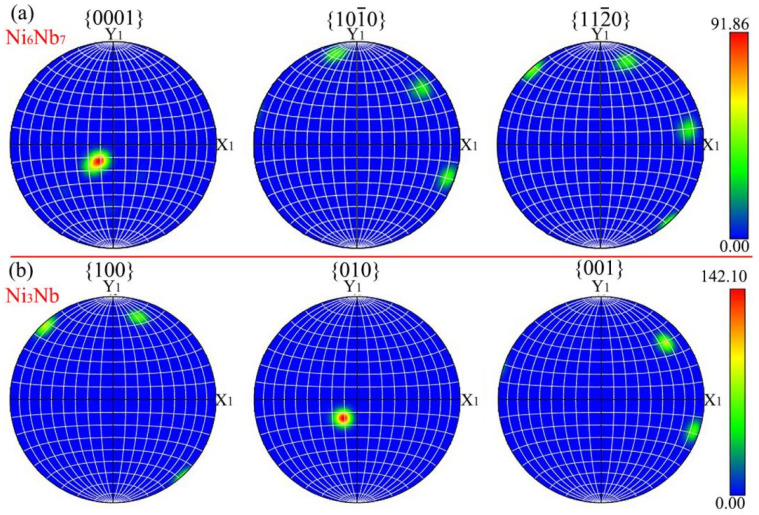
PFs: (**a**) Ni_6_Nb_7_; (**b**) Ni_3_Nb.

Figure 7a and Figure 7b respectively present the polar figures (PFs) of the Ni_6_Nb_7_ phase and the Ni_3_Nb phase in the area illustrated in Figure 6. Figure 7a depicts a prominent peak in the polar density in the vicinity of the {0001} PH, with the maximum intensity attaining 91.86. Based on the PFs shown in Figure 7a and the results presented in Figure 6, it can be deduced the Ni_6_Nb_7_ phase develops a fiber texture in the <0001> // growth direction (Z direction, the direction parallel to the welding interface). For the Ni_3_Nb phase, Figure 7b depicts a prominent peak in the polar density in the vicinity of the {010} PH, with the maximum intensity attaining 142.10. Similarly, it can be deduced the Ni_3_Nb phase develops a fiber texture in the <010> // growth direction.

**Figure 8:**
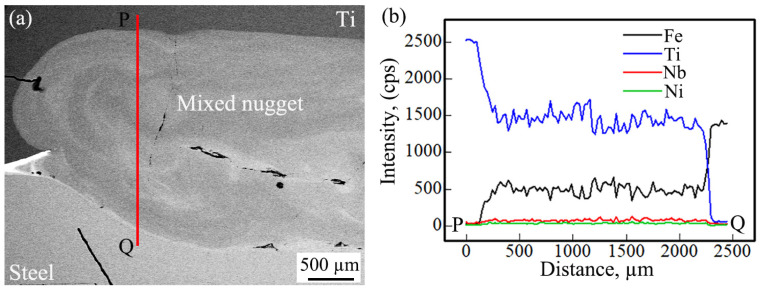
Low-magnification SEM and EDS results at the mixed nugget zone: (**a**) SEM image; (**b**) EDS results.

Figure 8a depicts the low-magnification SEM image of the nugget zone of the penetration-type joint. The Nb layer nor the Ni layer was observed in the mixed nugget. The EDS analysis along PQ in Figure 8a is presented in Figure 8b. The EDS results indicate that the components within the mixed nugget are distributed relatively uniformly on a macroscopic scale, primarily composed of Ti and Fe. The quantities of Ti and Fe detected in the mixed nugget are approximately in a fixed ratio, which suggests that Ti-Fe IMCs have been formed within the mixed nugget. Only a small quantity of Nb and Ni was detected in the mixed nugget. This is due to the fact that the applied interlayer of Nb and Ni was relatively thin in comparison to the base materials on both sides.

**Figure 9:**
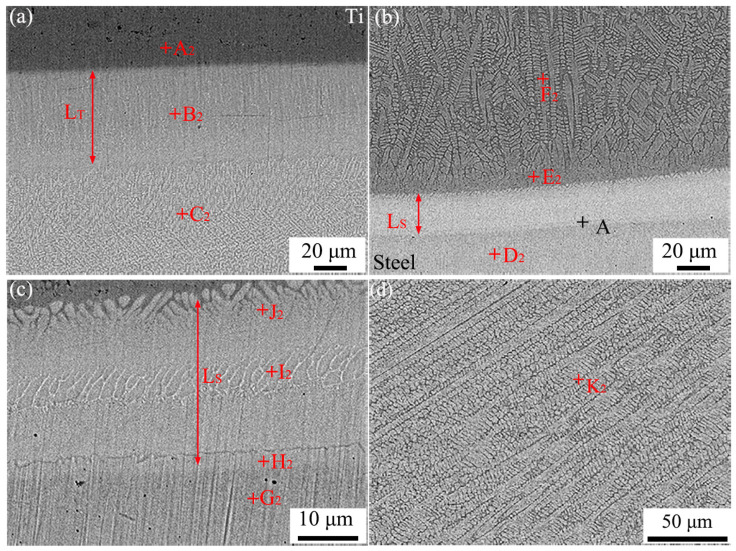
SEM images at the mixed nugget zone: (**a**) Ti side; (**b**) steel side; (**c**) enlarged image of A locations; (**d**) center zone.

Figure 9 presents the SEM image of the mixed nugget zone in the penetration-type joint. EDS analysis was carried out on the characteristic areas, and the results are presented in Table 5. Figure 9a presents the SEM image at the boundary of the mixed nugget adjacent to the Ti side. A layer-like structure (L_T_ layer) with a thickness of approximately 60 μm was observed at the edge of the mixed nugget. The compositional analysis revealed that the L_T_ layer (at point B_2_) contained a higher proportion of Fe compared to the adjacent Ti-nugget (at point A_2_), and its composition was a mixture of TiFe and α-Ti eutectic. The partially mixed nugget adjacent to the L_T_ layer is composed of dendritic grains. According to the EDS analysis results at point C_2_, it can be deduced that this area also comprises TiFe and α-Ti eutectic structure. However, this area contains a greater amount of TiFe phases than the L_T_ layer.

Figure 9b depicts the SEM image at the boundary of the mixed nugget adjacent to steel side. A layered structure (L_S_ layer) with a thickness of approximately 25 μm was also identified at the edge of the mixed nugget. A minor quantity of Ti, Nb, and Ni was detected in the steel in the vicinity of the L_S_ layer (at point D_2_). Figure 9c presents an enlarged view of the L_S_ layer, which was taken from point A in Figure 9b. The EDS analysis results suggest that the Ti content detected at H_2_ location is higher than that at G_2_ location and also surpasses the solubility of Ti in Fe. It can be inferred that the H_2_ area exhibits a coprecipitation structure of TiFe_2_ and α-Fe. At the locations of I_2_ and J_2_, the detected component compositions are in accordance with the stoichiometric numbers of the TiFe_2_ and TiFe phases, respectively. In Figure 9b, the component compositions at the locations of E_2_ and F_2_ are highly similar, both being co-precipitation structures of TiFe and α-Ti, with a relatively high TiFe content. Figure 9d depicts the SEM image of the central region of the mixed nugget. From this image, it can be observed that this region is mainly composed of coarse columnar crystals. The component analysis at location K_2_ reveals that the central region of the mixed nugget is also constituted by the TiFe and α-Ti eutectic structure. The TiFe presents a relatively hard phase. Mansor et al. reported that the hardness of the nugget of the RSWed joint composed of this phase could reach up to 1000 HV [23,24].

**Figure 10:**
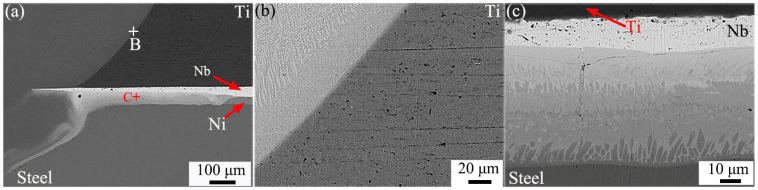
Interfacial SEM images of penetration-type joint: (**a**) edge of mixed nugget; (**b**) enlarged image of B location; (**c**) enlarged image of C location.

Figure 10 presents the SEM image of the interface zone the penetration-type joint. Figure 10a depicts the image of the junction between the mixed nugget and the interface region. It is evident that there remains a residual Nb layer at the interface outside the mixed nugget. Figure 10b,c present the magnified images of points B and C in Figure 10a. It can be observed that the structure at the lateral boundary of the mixed nugget is similar to the upper marginal structure depicted in Figure 9a. Meanwhile, the structure of the interface area outside the mixed nugget is similar to the interface area structure of the double-nugget-type joint shown in Figure 4f.

Therefore, when the bi-interlayer of Nb-Ni is utilized for RSW of Ti/steel, the residual interlayer in the joint persists continuously at the interface, which effectively prevented the mixing of the base metals on both sides when the resulting joint is of the double-nugget-type. In contrast, when the interlayer within the joint fractured, the metals on both sides intermixed, resulting in the formation of a mixed nugget primarily composed of Ti-Fe IMCs, and the interlayer lost its barrier function.

**Figure 11:**
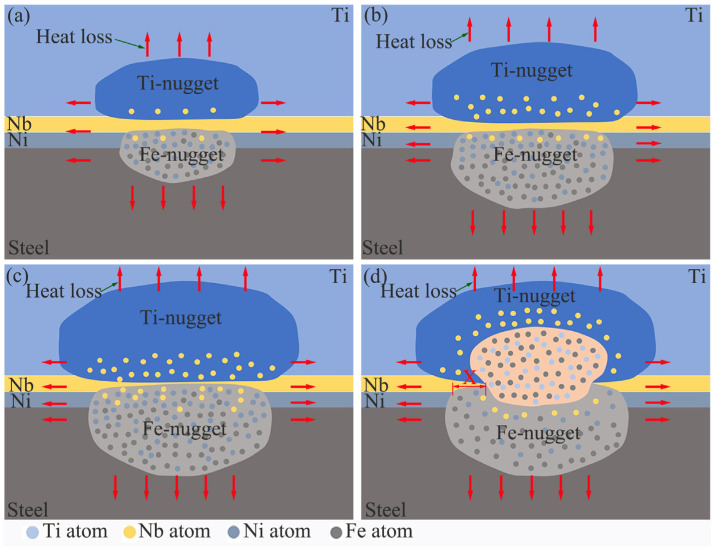
Schematic diagram of the formation of the nuggets under different welding heat inputs: (**a**) 9 kA; (**b**) 10 kA; (**c**) 11 kA; (**d**) 12 kA.

As previously mentioned, during the RSW of Ti/steel with a Nb-Ni bi-interlayer, the welding heat input varies, leading to different nugget patterns. Figure 11 presents the schematic diagram of the formation of the nuggets under different welding heat inputs. In this case, 0.04 mm-Ni + 0.05 mm-Nb is used as the interlayer, and the welding current is varied as an example. When the welding current is relatively low (e.g., 9 kA), the titanium, steel, and the Ni layer in the welding area gradually melt during the welding heating process. In this process, the molten nickel and steel blended. The Nb layer with a higher melting point did not melt; instead, it dissolved and mixed with the liquid metals on both sides. Simultaneously, Ti atoms diffused into the Nb. Since Ti and Nb can dissolve in each other, the Ti atoms remain in the Nb without diffusing across the interface to the other side. Conversely, Fe and Ni atoms also diffused into the Nb, but the solid Nb layer substantially reduced the cross-interface diffusion of Fe and Ni atoms towards the Ti side. After the power is cut off and the cooling process commences, the liquid metal on the titanium side formed a Ti-nugget primarily composed of α-Ti, while the liquid metal on the steel side formed a Fe-nugget primarily composed of α-Fe, as shown in Figure 11a. The red arrows in Figure 11 indicate the direction of heat dissipation from the molten core region during the cooling and solidification process. At the interface, IMCs such as Ni_3_Nb and Ni_6_Nb_7_ were formed. Increasing the welding current further, the high heat input caused an intensified dissolution of the Nb layer and an increase in the quantity of molten metal on both sides of the titanium and steel. This led to the formation of joints with a larger Ti-nugget and Fe-nugget, as well as a thinner residual Nb layer, as depicted in Figure 11b,c. The above situation indicates that the joint obtained under a lower welding heat input is of the double-nugget-type. Excessive welding heat input, such as a welding current of 12 kA, led to a relatively high peak temperature in the welding area. This caused the Nb layer in the welding zone to melt during the welding process. The melting of the Nb layer eliminated its barrier effect, and the liquid metals on both sides mixed. After cooling and solidification, the mixed liquid metal formed the mixed nugget, as shown in Figure 11d. In this scenario, the obtained joint is of a penetration-type. In the penetration-type joint, the mechanism of mixed nugget formation is similar to that of the fracture of the Nb layer during RSW of Ti/steel with Nb as the interlayer, which leads to the formation of a cross-interface nugget [1]. On the other hand, within the penetration-type joint, the mechanism governing the formation of IMCs at the interface adjacent to the residual Nb layer outer the mixed nugget (at location X in Figure 11d) is similar to that of the interface in the double-nugget-type joint.

**Figure 12:**
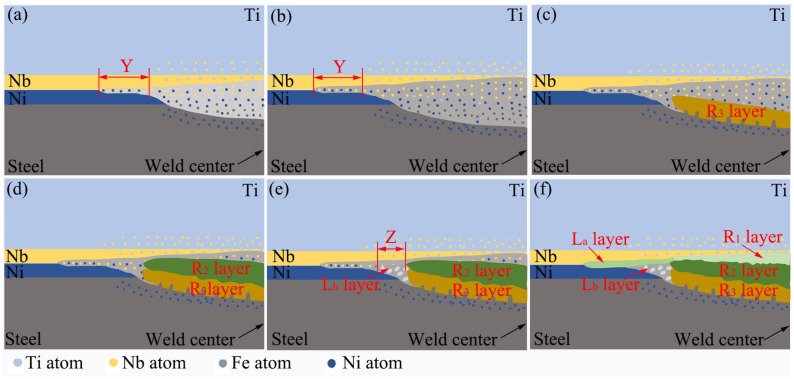
Schematic diagram depicting the formation process of the interfacial reaction layer: (**a**) initial stage of heating and melting; (**b**) later stage of heating and melting; (**c**) formation of the R_3_ layer; (**d**) formation of the R_2_ layer; (**e**) formation of the L_b_ layer; (**f**) formation of the R_1_ and L_a_ layers.

Figure 12 presents a schematic diagram depicting the formation process of the interfacial reaction layer of the double-nugget-type joint. When a lower welding current or a shorter welding time is employed for welding, the resultant joint is the double-nugget-type. As previously mentioned, during the heating process, a molten metal zone was formed on both the titanium side and the steel side. The molten metal on the steel side consists of the dissolved Ni and the melted steel as depicted in Figure 12a. During this process, the dissolution of Nb into the liquid metal on both sides and the diffusion of various elements took place. Meanwhile, at the outer edge of the weld, even though the heating temperature did not reach the melting point of Ni, atomic inter-diffusion took place at the closely contacting interface. Within a relatively narrow band (Y zone shown in Figure 12a), the contents of Nb and Ni reached the eutectic composition, and a eutectic liquid layer was formed when heated to approximately 1175 °C. As the heating process persisted, the melting regions on both sides expanded, the residual Nb layer thinned out, and simultaneously, the Y zone also extended outward, as depicted in Figure 12b. During the cooling process, apart from the formation of a nugget on the titanium side and another on the steel side, the reaction layers were also formed in the interface zone. Owing to the short duration of the welding heating, the composition distribution in the mixed liquid metal was non-uniform. When the nickel (Ni) content at the solidification front of the liquid metal is high on the steel side, the R_3_ layer composed of a solid solution of Ni in Fe is formed during the cooling process as depicted in Figure 12c. During the formation of the R_3_ layer, the solute atoms of Nb were directed towards the solidification front. When the temperature decreases to approximately 1400 °C, the concentrations of Ni and Nb at the solidification front also satisfy the stoichiometric ratio of Ni_3_Nb. The R_2_ layer composed of Ni_3_Nb was formed as depicted in Figure 12d. When the temperature decreases to approximately 1282 °C, as depicted in Figure 12e, the Z zone with a relatively higher Ni content at the periphery of the welding zone solidified to form a (Ni) + Ni_3_Nb eutectic structure (the dark-gray region within the L_b_ layer in Figure 4d). As the temperature continued to decline, the formation of additional Ni_3_Nb consumed a substantial quantity of Ni, resulting in the concentration of Ni in the mixed solution at the crystallization front failing to reach the critical value of the stoichiometric ratio of Ni_3_Nb. At this juncture, even though the reaction enthalpy of Ni_3_Nb is relatively low [25], the low concentration of Ni restricts its formation kinetically. As a result, the R_1_ layer composed of the Ni_6_Nb_7_ phase was produced, as depicted in Figure 12e. Meanwhile, within the Nb-rich region within the Z zone, a eutectic reaction occurred, leading to the formation of the Ni_3_Nb + Ni_6_Nb_7_ eutectic structure. Likewise, at the outer edge (zone Y) of the welding area, a eutectic reaction also occurred, resulting in the formation of the Ni_3_Nb + Ni_6_Nb_7_ eutectic structure as depicted in Figure 12e. In RSW, the heat dissipation along the electrodes caused the directional growth of Ni_3_Nb and Ni_7_Nb_6_ at the interface, which resulted in an anisotropic orientation. The Ni_3_Nb with an Orthorhombic structure presents a <010> crystal orientation parallel to the heat flow direction, whereas the Ni_7_Nb_6_ with a hexagonal structure demonstrates a <0001> crystal orientation parallel to the heat flow direction, thereby forming a preferred texture. After cooling and solidification, a eutectic mixture of Ni_3_Nb and Ni_6_Nb_7_ was formed.

RSW of Ti/steel, with copper foil [26,27] or nickel foil [28] employed as interlayer, resulted in the formation of a nugget across the interface within the joint. This nugget is predominantly composed of TiFe IMC [26,27,28]. In RSWed joint of Ti/steel with a Cu-Ni alloy interlayer, the formed nugget is composed of various IMCs, including those of the Ti-Fe, Ti-Ni, and Ti-Cu series [2,24]. In comparison with the aforementioned results, this study effectively prevented the mixing of molten base metals from both sides and inhibited the formation of Ti-Fe IMCs by utilizing Nb with a high melting point as an interlayer under appropriate welding parameter combinations. In this study, the Nb-Ni bi-interlayer was utilized to achieve solid joining between Ni and steel as well as between Nb and Ti in the joint. Meanwhile, only Ni-Nb IMCs were formed at the Ni/Nb interface, which laid a solid foundation for the further control of interfacial metallurgical reactions.

**Figure 13:**
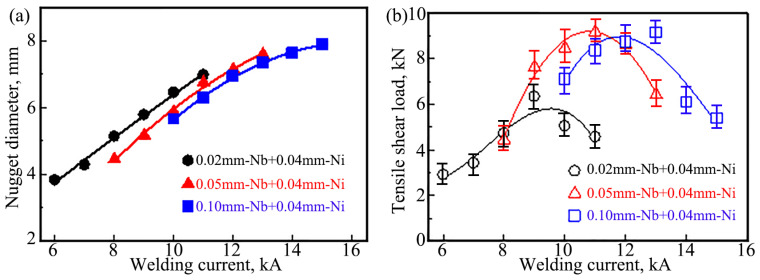
Effect of welding current on the nugget diameter and tensile shear load of the Ti/steel joint: (**a**) nugget diameter; (**b**) tensile shear load.

Figure 13 illustrates the impact of the welding current on the nugget diameter and the tensile shear load of the Ti/steel joint. The nugget diameter was measured on the fracture surface of the Ti side of the joint. As depicted in Figure 13a, for the joints featuring the interlayer of the three thickness combinations, the nugget diameter expanded as the welding current rose. An increase in the welding current results in an increase in generated heat, which leads to a greater volume of molten metal and subsequently forms a larger nugget. Under the same welding current conditions, when a thicker Nb layer was employed, the nugget diameter was marginally smaller. Since the thicker Nb layer improved heat conduction and dissipation, the effective heat input available for forming the nugget was reduced. Consequently, the nugget diameter decreased slightly as the thickness of the Nb layer increased.

As depicted in Figure 13b, for the joints with the interlayer of the three thickness combinations, the tensile shear load of the joints all exhibited a trend of initially increasing and subsequently decreasing with the increase in welding current. Here, the error bars denote the relative deviation of the tensile shear load data. The relative deviations of the tensile shear loads for the obtained joints are all less than 15%. When the thicknesses of the Nb layer were 0.02 mm, 0.05 mm, and 0.10 mm, the peak tensile shear load of the joint was achieved when the welding currents were 9 kA, 11 kA, and 13 kA, respectively. The corresponding peak tensile shear loads are approximately 5.35 kN, 8.18 kN, and 8.16 kN, respectively. This is primarily because the mode of Ti/steel joint has undergone a change. As previously mentioned, when the thicknesses of the applied Nb layers are 0.02 mm, 0.05 mm, and 0.10 mm, the joints obtained under the condition that welding currents less than 9 kA, 11 kA, and 13 kA respectively belong to the double-nugget-type. In this case, the nugget diameter is the primary factor influencing the tensile shear load of the Ti/steel joint. Within these ranges of welding current, as the welding current rose, the nugget diameter also expanded, resulting in an increase in the tensile shear load of the joint as the welding current increased. On the other hand, when the welding current surpasses the critical current, the joint obtained is the penetration-type joint. The mixed nugget in the penetration-type joint primarily consists of hard and brittle Ti-Fe IMCs, which results in a deterioration of the joint performance. As the welding current rose, the size of the formed mixed nugget expanded, and the proportion of the layered structure in the welding interface zone outside the mixed nugget decreased. Consequently, the tensile shear load of the joint decreased correspondingly.

When the applied Nb layer is of relatively large thickness, a higher heat input is necessary for the Nb layer to melt in the Ti/steel joint. Consequently, the critical welding current is higher. Therefore, the greater the thickness of the Nb layer employed, the larger the welding current at which the peak tensile shear load of the joint is attained. The nugget diameter of the joint welded at a higher critical welding current is larger. As a result, the peak tensile shear load of the joint welded with a 0.05 mm-thick Nb layer is significantly greater than that of the joint welded with a 0.02 mm-thick Nb layer. However, if a thicker Nb layer is utilized for welding, although the critical welding current can continue to rise, due to the excessive dissolution of Nb into the liquid metal during the welding process, it facilitates the formation of Ni_3_Nb and Ni_6_Nb_7_ IMCs. This led to a thicker IMCs layer in the interface area and impacted the joint performance. Therefore, in comparison to adding a 0.05 mm-thick Nb layer, the tensile shear load of the joint welded with a 0.10 mm-thick Nb layer does not exhibit a significant change.

**Figure 14:**
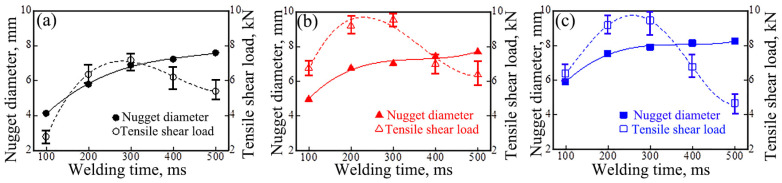
Effects of welding time on the nugget diameter and tensile shear load of joint: (**a**) 0.02 mm-Nb; (**b**) 0.05 mm-Nb; (**c**) 0.10 mm-Nb.

Figure 14 illustrates the influence of welding time on the nugget diameter and the tensile shear load of the joint. Here, the interlayer combinations in Figure 14a, Figure 14b and Figure 14c are 0.02 mm-Nb + 0.04 mm-Ni, 0.05 mm-Nb + 0.04 mm-Ni, and 0.10 mm-Nb + 0.04 mm-Ni respectively. The welding current selected is the welding current corresponding to the peak tensile shear load as depicted in Figure 13b. For the three types of joints with different interlayer combinations, the nugget diameter of the joints gradually increased as the welding time increased. The tensile shear load of all three types of joints demonstrated a pattern of initially increasing and then decreasing as the welding time increased. The relative deviations of the tensile shear loads for the obtained joints are all less than 15%. When the thicknesses of the Nb layer are 0.02 mm, 0.05 mm, and 0.10 mm respectively, the tensile shear load of the joints reached its peak at a welding time of 300 ms, with values of 6.2 kN, 8.7 kN, and 8.4 kN respectively. The nugget diameter and the tensile shear load of the joint vary with the prolongation of the welding time as depicted in Figure 14. The reason for this is analogous to the influence of the welding current on them. It is noteworthy that the peak tensile shear load of the joint obtained herein is slightly higher than that of the similar joint in Figure 13b. This is due to the fact that when the welding current was varied, the welding time was fixed at 200 ms; when the welding time was varied, the welding current was the critical current shown in Figure 13b, whereas the welding time for the joint in Figure 11 to reach the peak tensile shear load was 300 ms.

Q235 steel and titanium plates with a thickness of 2 mm were joined through RSW with an interlayer of 0.04 mm-thick nickel foil; the maximum tensile shear load of the joint reached 8.0 kN [21], whereas the maximum tensile shear load of the joint was 3.7 kN when 1 mm-thick 316L stainless steel and TC4 alloy plates were welded by RSW with an interlayer of 0.03 mm-thick nickel foil [28]. In the RSW of Ti/Q235 with an interlayer of 0.3 mm thick Nb foil, the maximum tensile shear load of 6.17 kN was achieved [20]. The maximum tensile shear load of the joint achieved through direct RSW between Q235 steel and titanium plates with 1 mm thickness was approximately 3.9 kN [1]. In comparison with the aforementioned results, the maximum tensile shear load of the joint acquired in this study is higher, which suggests that the utilization of a Nb-Ni bi-interlayer to regulate the RSW interface of Ti/steel is a viable approach.

**Figure 15:**
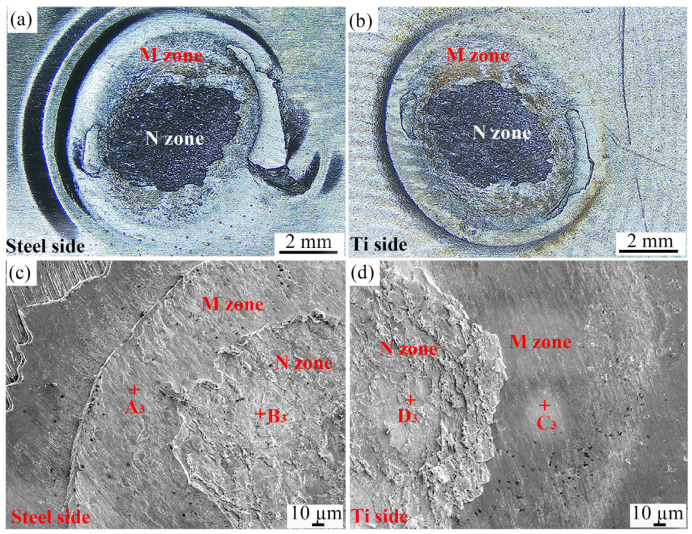
Fractures of double-nugget-type joint: (**a**) steel side; (**b**) Ti side of joint; (**c**) locally magnified view of steel side; (**d**) locally magnified views of Ti side.

In this study, all the joints obtained failed due to interfacial tearing during the tensile shear testing. Figure 15a and Figure 15b respectively present the typical fracture images of the steel side and Ti side of the double-nugget-type joint. Based on the fracture morphology characteristics, the fracture surfaces on both sides of the joint can be categorized into a dark zone (N zone) at the weld center and a ring-shaped zone (M zone) at the periphery of the weld. As illustrated, the surface of the N zone demonstrates roughness, while the surface of the M zone is relatively smooth.

Figure 15c and Figure 15d respectively present the locally magnified views of the fracture surfaces on the steel side and the Ti side. A component analysis of the characteristic regions was carried out, and the results are presented in Table 6.

On the fracture surfaces of the N zone on both sides, numerous tear ridges are present. Nevertheless, certain cleavage steps can be observed in the M zone on the steel side, whereas, only a small number of furrows and slight deformation traces are distributed on the surface of the M zone on the Ti side. Based on the EDS results of the N zones (at locations B_3_ and D_3_) on both sides of the fracture, it can be deduced that the joint failure in the N zone occurred at the Ni-Nb IMCs layer. Based on the EDS results of the M zones (at A_3_ and C_3_) on both sides of the fracture, it can be deduced that the joint failure in the M zone occurred at the interface between Ti and Nb. The former can be attributed to the formation of a relatively thick Ni_6_Nb_7_ and Ni_3_Nb layer at the interface zone; the latter is due to the inadequate heating at the periphery of the weld, which led to the failure of Ti and Nb to form a firm bond.

**Figure 16:**
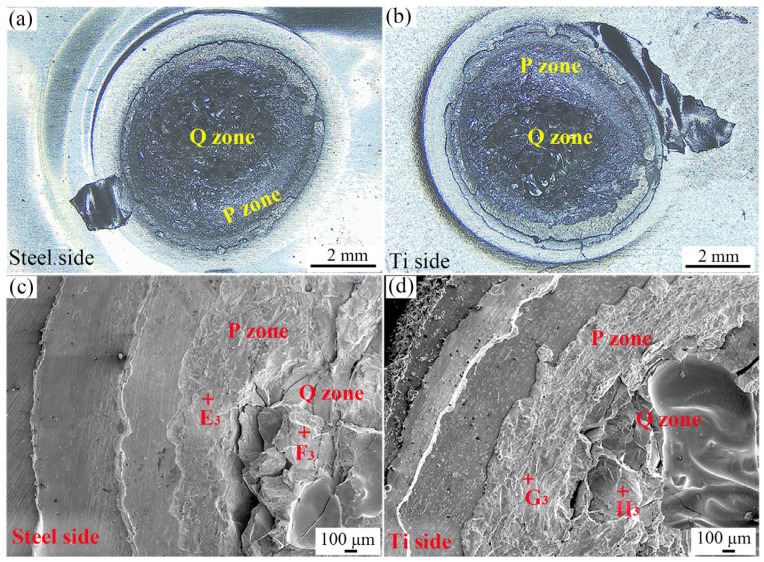
Fractures of penetration-type joint: (**a**) steel side; (**b**) Ti side of joint; (**c**) locally magnified view of steel side; (**d**) locally magnified views of Ti side.

Figure 16a and Figure 16b respectively present the typical fracture images of the steel side and Ti side of the penetration-type joint.

Based on the fracture morphology characteristics, the fracture surfaces on both sides of the joint can be classified into a Q zone at the weld center and a P zone at the periphery of the weld. In comparison with the P zone, not only deeper tear ridges but also cross-sections of smooth-walled holes were observed in the Q zone.

Figure 16c and Figure 16d respectively present the locally magnified views of the fracture surfaces on the steel side and the Ti side. The EDS results at the characteristic regions are also presented in Table 6. Based on the EDS results of the Q zones (at locations F_3_ and H_3_) on both sides of the fracture, it can be deduced that the joint failure in the Q zone occurred at the Ti-Fe IMCs layer. Based on the EDS results of the P zones (at E_3_ and G_3_) on both sides of the fracture, it can be deduced that the joint failure in the P zone occurred at the Ni-Nb IMCs layer.

When conducting RSW of titanium and Q235 steel plates with a Nb-Ni bi-interlayer, the Nb layer serves as a crucial barrier function. Therefore, there is a compatibility between the selected welding parameters and the thickness of the interlayer. Based on the nugget patter and the tensile shear load of the joint, the optimal parameter combinations obtained in this study for the selected interlayers of 0.02 mm-Nb + 0.04 mm-Ni, 0.05 mm-Nb + 0.04 mm-Ni, and 0.10 mm-Nb + 0.04 mm-Ni are 9 kA welding current–300 ms welding time, 11 kA welding current–300 ms welding time, and 13 kA welding current–300 ms welding time, respectively.

When the obtained joint is of the double-nugget-type, the failure of the joint primarily occurs in the relatively thick Ni_7_Ni_6_ and Ni_3_Nb layers in the interface zone. When the obtained joint is of the penetration-type, the failure of the joint progresses from the Ni-Nb IMCs layer at the periphery of the weld to the mixed nugget composed of Ti-Fe IMCs at the center zone of the weld. Therefore, when the bi-interlayer of Nb-Ni is employed for RSW of Ti/steel, the IMCs formed at the welding interface remain the primary factor constraining the performance of the joints. In subsequent research, it is imperative to concentrate on ameliorating the distribution of IMCs in the interface zone in order to further improve the performance of the joint.

## 4. Conclusions

Through this study, the following conclusions can be drawn:

1. When a bi-interlayer of Nb-Ni is used for the RSW of Ti/steel, the joints obtained display double-nugget-type and penetration-type joints. The critical welding current required to transition from double-nugget- to mixed-type joint increases with increasing Nb interlayer thickness.
2. For the double-nugget-type joint, a continuous residual Nb layer is present at the welding interface zone. In the region between the residual Nb layer and the steel, Ni_6_Nb_7_ and Ni_3_Nb layers have been formed.
3. For the penetration-type joint, a mixed nugget composed of Ti-Fe IMCs was formed at the center zone of the weld.
4. Under the condition of employing a bi-interlayer consisting of 0.05 mm-Nb and 0.04 mm-Ni, the tensile shear load of the joint attained the maximum value of approximately 8.7 kN when the welding current was 11 kA, the welding time was 300 ms, and the electrode pressure was 3 kN.
5. For the interlayer combination of 0.10 mm Nb + 0.04 mm Ni, the optimal welding parameters were 13 kA and 300 ms.
6. The double-nugget-type joint failure primarily occurred in the Ni-Nb IMCs layer, whereas the penetration-type joint failure took place in the Ni-Nb IMCs layer situated at the periphery of the weld and the mixed nugget composed of Ti-Fe IMCs in the central zone of the weld. Consequently, the peak tensile-shear load is determined by the competition between nugget size (beneficial) and the thickness of brittle IMC layers (detrimental).

## Figures and Tables

**Table 1:** Chemical compositions of the experimental materials (mass fraction, %).

	C	N	H	O	Fe	Mn	P	S	Si	V	Ti
TA2	0.01	0.02	0.002	0.14	0.07	-	-	-	-	-	Bal.
Q235	0.14	-	-	-	Bal.	1.0	0.04	0.02	0.4	0.06	-

**Table 2:** Welding conditions (changing welding current).

	Series 1	Series 2	Series 3
Nb foil thickness (mm)	0.02	0.05	0.10
Welding current (kA)	6~11	8~13	10~15
Welding time (ms)	200	200	200
Electrode force (kN)	3	3	3

**Table 3:** Welding conditions (changing welding time).

	Series 4	Series 5	Series 6
Nb foil thickness (mm)	0.02	0.05	0.10
Welding current (kA)	9	11	13
Welding time (ms)	100~500	100~500	100~500
Electrode force (kN)	3	3	3

**Table 4:** EDS results of each location marked in Figure 4 (at.%).

	A_1_	B_1_	C_1_	D_1_	E_1_	F_1_	G_1_	H_1_	I_1_	J_1_
Ti	0.0	3.0	0.0	0.0	0.0	0.0	1.0	0.5	0.2	0.0
Nb	32.9	93.8	0.0	33.8	34.9	18.3	55.8	26.6	10.1	0.5
Ni	67.1	3.2	98.4	63.5	59.8	81.7	41.5	68.8	37.9	1.3
Fe	0.0	0.0	1.6	0.0	5.3	0.0	1.7	4.1	51.8	98.2

**Table 5:** EDS results of each location marked in Figure 8 (at.%).

	A_2_	B_2_	C_2_	D_2_	E_2_	F_2_	G_2_	H_2_	I_2_	J_2_	K_2_
Ti	99.1	70.0	61.4	1.3	51.9	53.6	1.8	8.7	28.7	40.0	57.2
Nb	0.1	1.6	1.4	0.3	0.7	0.5	0.0	0.1	1.2	1.1	0.7
Ni	0.2	1.1	1.9	0.2	2.2	2.0	0.1	0.4	0.0	1.4	2.8
Fe	0.6	27.3	35.3	98.2	45.2	43.9	98.1	90.8	70.1	57.5	39.3

**Table 6:** EDS results of each location marked in Figure 15 and Figure 16 (at.%).

	A_3_	B_3_	C_3_	D_3_	E_3_	F_3_	G_3_	H_3_
Ti	0.4	0.2	96.7	0.9	0.0	44.5	0.0	53.1
Nb	98.1	53.5	2.5	58.0	57.5	8.7	54.6	2.0
Ni	1.5	38.0	0.5	32.8	31.7	3.8	33.2	4.3
Fe	0.0	8.3	0.3	8.3	10.8	43.0	12.2	40.6

## Data Availability

The original contributions presented in this study are included in the article. Further inquiries can be directed to the corresponding author.
